# The 20 years transition of clinical characteristics and metabolic risk factors in primary liver cancer patients from China

**DOI:** 10.3389/fonc.2023.1109980

**Published:** 2023-03-14

**Authors:** Yezhou Ding, Mingyang Feng, Di Ma, Gangde Zhao, Xiaolin Wang, Baoyan An, Yumin Xu, Shike Lou, Lanyi Lin, Qing Xie, Kehui Liu, Shisan Bao, Hui Wang

**Affiliations:** ^1^ Department of Infectious Diseases, Ruijin Hospital, Shanghai Jiao Tong University School of Medicine, Shanghai, China; ^2^ Department of General Surgery, Ruijin Hospital, Shanghai Jiao Tong University School of Medicine, Shanghai, China

**Keywords:** clinical traits, fetoprotein, alanine transaminase, glycolipid, liver cancer

## Abstract

**Background:**

The clinical characteristics of primary liver cancer (PLC) patients are changing, maybe due to hepatitis viral vaccination and lifestyle changes, etc. The linkage between these changes and outcomes among these PLCs has not yet been fully elucidated.

**Methods:**

It was identified total of 1691 PLC cases diagnosed between 2000 ~ 2020. Cox proportional hazards models were utilized to determine the connections between the clinical presentations and their close risk factor(s) from PLC patients.

**Results:**

The average age of PLC patients increased gradually from 52.74 ± 0.5 years in 2000 ~ 2004 to 58.63 ± 0.44 years in 2017 ~ 2020, accompanied by an increased proportion of females from 11.11% to 22.46%, and non-viral hepatitis-related PLC was raised from 1.5% to 22.35%. 840 (49.67%) PLC patients with alpha-fetoprotein (AFP) < 20ng/mL (AFP-negative). The mortality was 285 (16.85%) or 532 (31.46%) PLC patients with alanine transaminase (ALT) between 40 ~ 60 IU/L or ALT > 60 IU/L. The PLC patients with pre-diabetes/diabetes or dyslipidemia also increased from 4.29% or 11.1% in 2000 ~ 2004 to 22.34% or 46.83% in 2017 ~ 2020. The survival period of the PLC patients with normoglycemia or normolipidemic was 2.18 or 3.14 folds longer than those patients with pre-diabetes/diabetes or hyperlipidemia (P<0.05).

**Conclusions:**

It was gradually increased that age, the proportion of females, non-viral hepatitis-related causes, AFP-negative, and abnormal glucose/lipids among PLC patients. Proper control of glucose/lipids or ALT may improve the prognosis of PLCs.

## Introduction

Primary liver cancer (PLC) is the fourth leading cause of cancer-related death worldwide (1). Hepatocellular carcinoma (HCC) is the most common (>80%) type of PLC (1–3). There are quite distinct risk factors contributing to the development of PLC at genetic and molecular levels between Chinese and Causations (4). The main risk factor for HCC in Chinese or Causations is chronic hepatitis B virus (HBV) infection or hepatitis C virus (HCV) infection, as well as, alcohol intake, respectively. The causes of PLC in China include chronic infection with HBV and/or HCV, excessive alcohol consumption, non-alcoholic fatty liver disease (NAFLD), and aflatoxin exposure (5, 6). The incidence of PLC has been improved following lifestyle changes, i.e. quitting smoking, reducing alcohol, and high-sugar/fat diet consumption. The reduced morbidity of PLC is also related to the introduction of new anti-viral therapies, nucleos(t)ide analogs (NAs), and direct-acting antivirals (DAAs) (1, 7) against HBV or HCV infections over the last two decades, contributing to the significant change of clinical characteristics of PLC.

Dietary intake has changed substantially following rapid economic development over the last decades in China, i.e. significantly reduced grain intake, but increased fat intake (8). In addition, daily salt intake is also much higher, but vegetables and/or fruits are lower than recommended (9). All of these factors could contribute to the rapidly rising prevalence of metabolic syndrome, i.e. up to 24.2% from a large Chinese cross-sectional study (10). Thus, metabolic syndrome, characterized by visceral obesity, dyslipidemia, hyperglycaemia, and hypertension, is a major challenge in public health nationwide (9, 11, 12). Furthermore, disturbed glycemia is associated with an increased risk of PLC (13). However, it remains to be explored that the potential role of dyslipidemia and hyperglycaemia involves the development of PLC.

We investigated the epidemiology of PLC over the last 20 years, including age, sex, causes, metabolic risk factors, and prognosis of PLC patients. Such information may offer novel therapeutic management to improve outcomes.

## Materials and methods

### Patients

**Figure 1:**
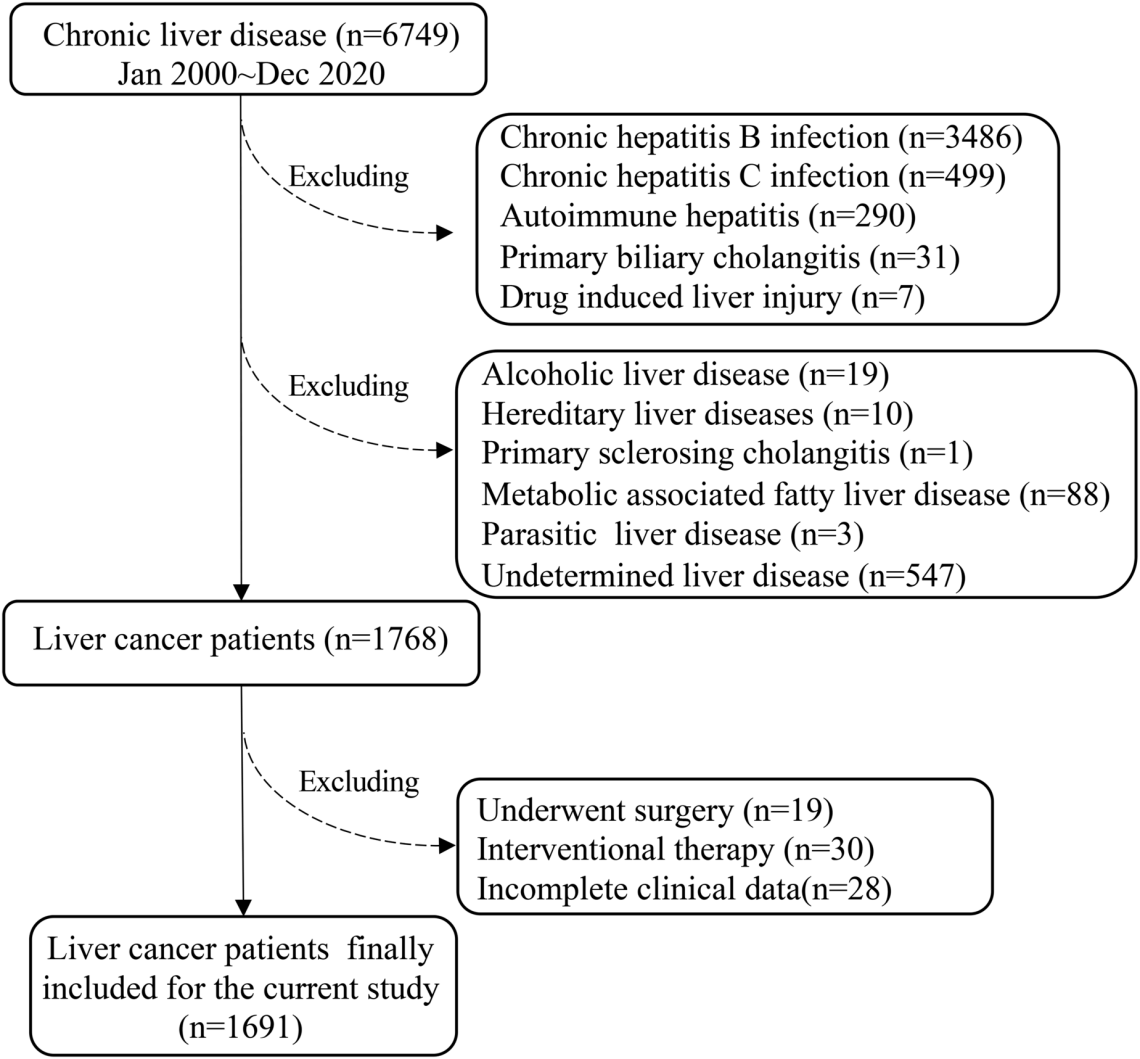
The flow chart demonstrated the selection criteria for PLC patients.

It was performed a cohort retrospective study of PLC in the Ruijin Hospital (a tertial teaching hospital, Shanghai Jiao Tong University School of Medicine, China) over the last 20 years from January 2000 to December 2020. The total number of chronic liver diseases identified in The Department of Infectious Diseases, Ruijin Hospital were 6749 (**Figure 1**). Subsequently, 4981 were excluded: chronic HBV infection (n=3486), chronic HCV infection (n=499), autoimmune hepatitis (AIH) (n=290), primary biliary cholangitis (PBC) (n=31), drug-induced liver injury (DILI) (n=7), alcoholic liver disease (ALD) (n=19), hereditary liver disease (n=10), primary sclerosing cholangitis (PSC) (n=1), NAFLD (n=88), parasitic liver disease (PLD) (n=3), undetermined (n=547) (**Figure 1**).

Based on *the American Association for the Study of Liver Diseases* (*AASLD) Guidelines for the management of HCC* (14), there were 1768 PLC cases in the current study fitted with the inclusion criteria, i.e.: only diagnosed PLC with treatment-naïve and complete clinical/laboratory data at the time of diagnosis. Subsequently, 77 cases were further excluded, because these patients received surgery (n=19) or interventional therapy (n=30), incomplete data (n=28). Thus, the final eligible PLC patients for the current study were 1691 (**Figure 1**).

### Definitions and data assessment

The definition of CHB was based on AASLD 2018 Hepatitis B Guidance, i.e. persistent presence of HBsAg for more than six months (15). The definition of CHC was based on AASLD 2019 Hepatitis C Guidance (16),, i.e. persistent detected HCV RNA for more than six months.

The 1691 cases were subclassed into normoglycemia (3.9 ~ 5.5mmol/L), and pre-diabetes/diabetes (≥ 5.5 mmol/L) groups, based on the latest guidelines of the American Diabetes Association (17).

In addition, these 1691 cases were also classified into normolipidemic and dyslipidemia groups, based on the Chinese guidelines for the prevention and treatment of dyslipidemia in adults, i.e: the cut-off: total cholesterol (TC) ≥ 5.2 mmol/L, low-density lipoprotein cholesterol (LDL-C) ≥ 3.4 mmol/L (130 mg/dl), triglycerides (TG) ≥ 1.7 mmol/L and apolipoprotein B (ApoB) ≥ 1.13 g/L. The patients with cut-off values below the above were classified as the normolipidemic group.

The Barcelona clinic liver cancer (BCLC) stage, the model for end-stage liver disease (MELD) score, albumin-bilirubin (ALBI) grade, and platelet-albumin-bilirubin (PALBI) Grade is followed by previous publication (18–21).

### Statistical analysis

All statistics were performed, using SPSS version 22.0 software (SPSS Inc., Chicago, IL, USA). Data were presented as mean ± standard derivation for normally distributed continuous data, as median (interquartile range, Q25*–*Q75) for abnormally distributed continuous data, or as actual values for categorical data. Baseline characteristics were summarized, using descriptive statistics. Groups were compared using χ^2^ tests for categorical and Mann-Whitney U tests for continuous variables. Multivariable Cox’s proportional hazard model was carried out to identify independent factors associated with the outcome (death) through time and cumulative overall survival (OS) was calculated to estimate the hazard ratios. Factors with p < 0.05 in the univariate cox regression analysis were entered into the multivariable model. Survival rates were estimated by the Kaplan-Meier method and the differences were compared using the log-rank test. *P* value < 0.05 was considered to indicate a statistically significant difference.

## Results

### Baseline clinical characteristics of PLC patients

A total of 1691 PLC patients were included in the current study, i.e. 1605 were HCC (94.91%), 58 were intrahepatic cholangiocarcinoma (ICC) (3.42%), and 28 were HCC-ICC mixed types (1.66%). There was significant difference of the average age between HCC and ICC (56.62 ± 18.51 vs 60.8 ± 10.3 years, *P* < 0.05). Among 1183/1691 (69.96%) PLC patients with cirrhosis, 97.38% or 1.86% were HCC or ICC (*P* < 0.05). There were 93.44% or 6.56% BCLC grade A-C or D; 91.01%; or 8.99% were Child-Turcotte-Pugh class A-B or stage C. The MELD score, ALBI score, alpha-fetoprotein (AFP), aspartate aminotransferase (AST); and total bilirubin (Tbil) level were significantly higher in HCC patients compared to ICC patients (*P* < 0.05). In contrast, albumin (Alb) levels, neutrophils, monocytes, lymphocytes and platelet counts from the HCC patients were significantly lower than the ICC patients (*P* < 0.05). There was no significant difference in survival time between HCC and ICC patients (*P* < 0.05) (**Table 1**).

**Table 1:** Clinical characteristics of PLC patients.

Charcteristics	Total (n, %)	HCC (n, %)	ICC (n, %)	P value
Number	1691 (100%)	1605 (94.91%)	58 (3.42%)	
Age, years	56.8 ± 18.14	56.62 ± 18.51	60.8 ± 10.3	<0.05
Gender				<0.05
Male	1360 (80.43%)	1312 (96.47%)	33 (2.43%)	
Female	331 (19.57%)	293 (88.52%)	25 (7.55%)	
Cirrhosis				<0.05
No	508 (30.04%)	453 (89.17%)	36 (7.09%)	
Yes	1183 (69.96%)	1152 (97.38%)	22 (1.86%)	
BCLC stage				0.6
A	573 (33.89%)	548 (95.64%)	22 (3.84%)	
B	510 (30.16%)	490 (96.08%)	15 (2.94%)	
C	497 (29.39%)	476 (95.77%)	19 (3.82%)	
D	111 (6.56%)	91 (81.98%)	2 (1.8%)	
Child-Pugh class				0.13
A	1086 (64.22%)	1047 (96.41%)	39 (3.59%)	
B	453 (26.79%)	432 (95.36%)	18 (3.97%)	
C	152 (8.99%)	126 (82.89%)	1 (0.66%)	
MELD score	7.27 (6.93∼7.61)	7.48 (7.12∼7.83)	4.3 (3.1∼5.49)	<0.05
ALBI score	-1.99 (-2.03∼1.96)	-1.98 (-2.02∼-1.94)	-2.2 (-2.37∼-2.02)	<0.05
pALBI score	-2.13 (-2.15∼-2.1)	-2.12 (-2.14∼-2.1)	-2.19 (-2.32∼-2.05)	0.19
AFP (ng/mL)	2173 (1895∼2452)	2284 (1991∼2576)	452.4 (215.2∼1120)	<0.05
ALT (IU/L)	63.46 (57.13∼69.79)	63.73 (57.09∼70.37)	61.93 (42.38∼81.48)	0.43
AST (IU/L)	83.54 (75.83∼91.25)	84.59 (76.63∼92.56)	54.42 (35.07∼73.77)	<0.05
Alb (g/L)	34.34 (33.98∼34.7)	34.19 (33.82∼34.57)	36.02 (34.43∼37.6)	<0.05
Tbil (μmol/L)	44.35 (40.4∼48.31)	45.08 (40.95∼49.21)	36.54 (19.97∼53.12)	<0.05
Neutrophil count (× 10^9^/L)	3.85 (3.64∼4.06)	3.82 (3.61∼4.04)	4.3 (3.71∼4.89)	<0.05
Monocyte count (× 10^9^/L)	0.5 (0.47∼0.52)	0.49 (0.47∼0.52)	0.53 (0.46∼0.59)	<0.05
Lymphocyte count (× 10^9^/L)	1.47 (1.31∼1.62)	1.46 (1.3∼1.62)	1.54 (1.39∼1.69)	<0.05
Platelet count (× 10^9^/L)	138.9 (134.7∼143.1)	138.9 (134.7∼143.1)	186 (164.2∼207.7)	<0.05
Blood glucose (mmol/L)	5.58 (5.47∼5.69)	5.58 (5.47∼5.69)	5.59 (5.17∼6.01)	0.42
TG (mmol/L)	1.16 (1.11∼1.22)	1.16 (1.1∼1.22)	1.2 (1∼1.4)	0.12
TC (mmol/L)	4.02 (3.91∼4.12)	4.01 (3.9∼4.12)	4.27 (3.83∼4.71)	0.27
LDL (mmol/L)	2.41 (2.33∼2.5)	2.41 (2.32∼2.5)	2.55 (2.22∼2.88)	0.31
ApoB (mmol/L)	0.83 (0.8∼0.86)	0.83 (0.8∼0.86)	0.82 (0.73∼0.9)	0.58
OS (months)	24.98 (23.38∼26.59)	25.32 (23.66∼26.99)	17.14 (12.76∼21.53)	0.09

BCLC stage, the Barcelona Clinic Liver Cancer stage; MELD, end-stage liver disease; ALBI, albumin-bilirubin; pALBI, platelet-albumin-bilirubin; AFP, alpha-fetoprotein; ALT, alanine aminotransferase; AST, aspartate aminotransferase; Alb, albumin; Tbil, total bilirubin; TG, triglycerides; TC, total cholesterol; LDL-C, low-density lipoprotein cholesterol; ApoB, apolipoprotein B; OS, overall survival.

Chronic hepatitis B (CHB) was still the predominant aetiology (83.32%) (**Figure S1A**). The percentage of antiviral treatment among CHB-related PLC patients with or without cirrhosis was 762/1183 (64.41%) and 115/508 (22.64%), respectively (**Figure S1B**). The proportion of CHB-related PLC patients receiving antiviral therapy was raised from 24.11% in 2000 ~ 2004 to 63.45% in 2017 ~ 2020 (**Figure S1C**).

### Changes in clinical characteristics of PLC patients over 20 years

**Figure 2:**
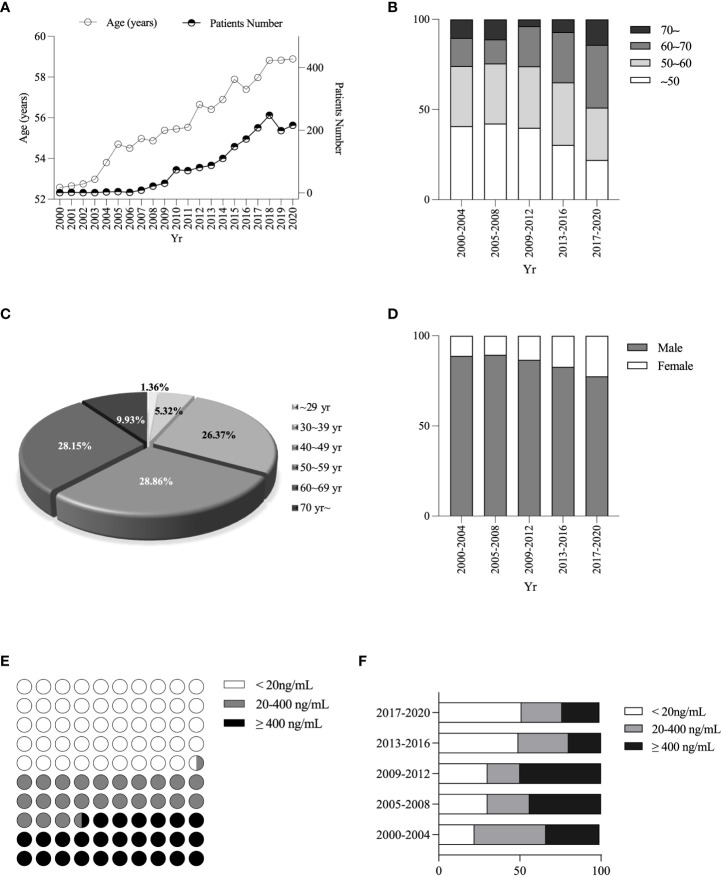
Changes in age, sex, and different AFP levels ratio among PLC patients. Changes in age of diagnosis of PLC patients **(A)**. Changes in age composition ratio **(B)**. Percentage of PLC patients’ age **(C)**. Changes in sex ratio **(D)**. Stratification of AFP levels **(E)**. Changes in the proportion of different AFP stratifications **(F)**.

The average age of PLC patients increased gradually from 52.74 ± 0.5 to 58.63 ± 0.44 years over the last two decades (**Figure 2A**). Furthermore, the percentage of patients over 50 years increased from 59.23% in 2000 ~ 2004 to 77.98% in 2017 ~ 2020 (**Figure 2B**). The age distribution of PLC patients was 488/1691 (28.86%), 476/1691 (28.15%), 446/1691 (26.37%) or 281/1691 (16.62%) for 50 ~ 59, 60 ~ 69, 40 ~ 49, or the remaining age group (**Figure 2C**). The percentage of males decreased from 88.89% in 2000 ~ 2004 to 77.54% in 2017 ~ 2020, while the females increased from 11.11% to 22.46% (**Figure 2D**). PLC patients with alpha-fetoprotein (AFP) < 20ng/mL, 20ng/mL ≤ AFP < 400ng/mL, or AFP ≥ 400 ng/mL was 840/1691 (49.67%), 403/1691 (23.83%) or 448/1691 (26.5%), respectively (**Figure 2E**), and the proportion of patients with AFP < 20ng/mL (AFP-negative) gradually was increased from 24.2% in 2000 ~ 2004 to 51.43% in 2017 ~ 2020 (**Figure 2F**).

**Figure 3:**
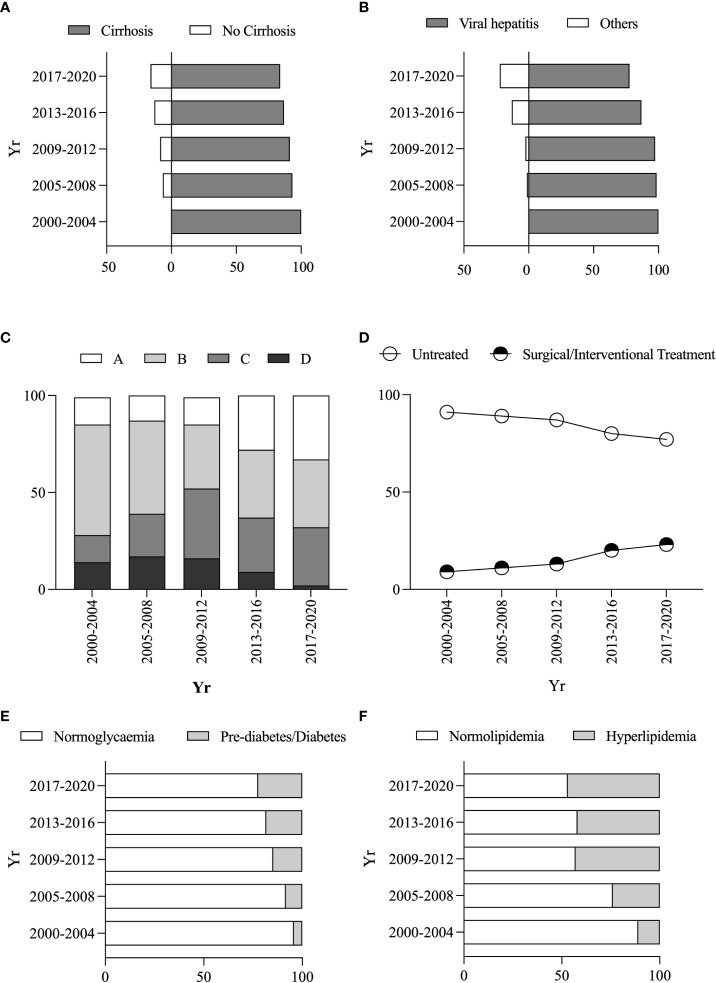
Changes in clinical characteristics and metabolic risk factors of PLC patients. Changes in the proportion of combined *vs* without cirrhosis **(A)**. Changes in the proportion of non-/viral hepatitis-related PLC patients **(B)**. Changes in the proportion of different BCLC stages **(C)**. Changes in the proportion of different treatment protocols **(D)**. Changes in the proportion of pre-diabetes/diabetes *vs* normoglycemia **(E)**. Changes in the proportion of hyperlipidemia *vs* normolipidemia **(F)**.

The proportion of PLC patients with cirrhosis decreased from 93.37% in 2005 ~ 2008 to 83.86% in 2017 ~ 2020 (**Figure 3A**), while those with non-viral hepatitis-related PLC increased from 1.5% in 2005 ~ 2008 to 22.35% in 2017 ~ 2020 (**Figure 3B**). The proportion of patients with BCLC A stage increased from 14.29% in 2000 ~ 2004 to 33.01% in 2017 ~ 2020 (**Figure 3C**), and the proportion of the patients who received any treatment (including surgical or interventional therapy) increased from 9.5% in 2000 ~ 2004 to 23.12% in 2017 ~ 2020 (**Figure 3D**).

The percentage of patients with Child-Pugh class A or MELD score ≤ 14 increased from 28.57% or 40% in 2000 ~ 2004 to 71.33% or 93.04% in 2017 ~ 2020 (**Figures S2A, B**), and the proportion of ALBI grade I or pALBI grade I was raised from 13.39% or 17.24% in 2005 ~ 2008 to 28.12% or 58.76% in 2017 ~ 2020, respectively (**Figures S2C, D**).

### Metabolic risk factors and related prognosis among PLC patients

Within these PLC patients, 866 (52.3%), 313 (18.51%), 712 (42.11%) or 979 (57.89%) had normoglycemia, pre-diabetes/diabetes, normolipidemic or hyperlipidaemia. The PLC patients with elevated blood glucose (blood glucose reaching pre-diabetes/diabetes levels) increased from 4.29% in 2000 ~ 2004 to 22.34% in 2017 ~ 2020 (**Figure 3E**). Similarly, the PLC patients with dyslipidaemia increased from 11.1% in 2000-2004 to 46.83% in 2017-2020 (**Figure 3F**). There were increased percentages of female patients with elevated blood glucose and hyperlipidaemia from 12.5% to 21.24% and 14.29% to 23.9% from the period of 2005 ~ 2008 to the period of 2017 ~ 2020, whereas the ratio of male patients declined from 87.5% or 85.71% to 76.1% or 78.76% (**Figure S3A, B**).

**Figure 4:**
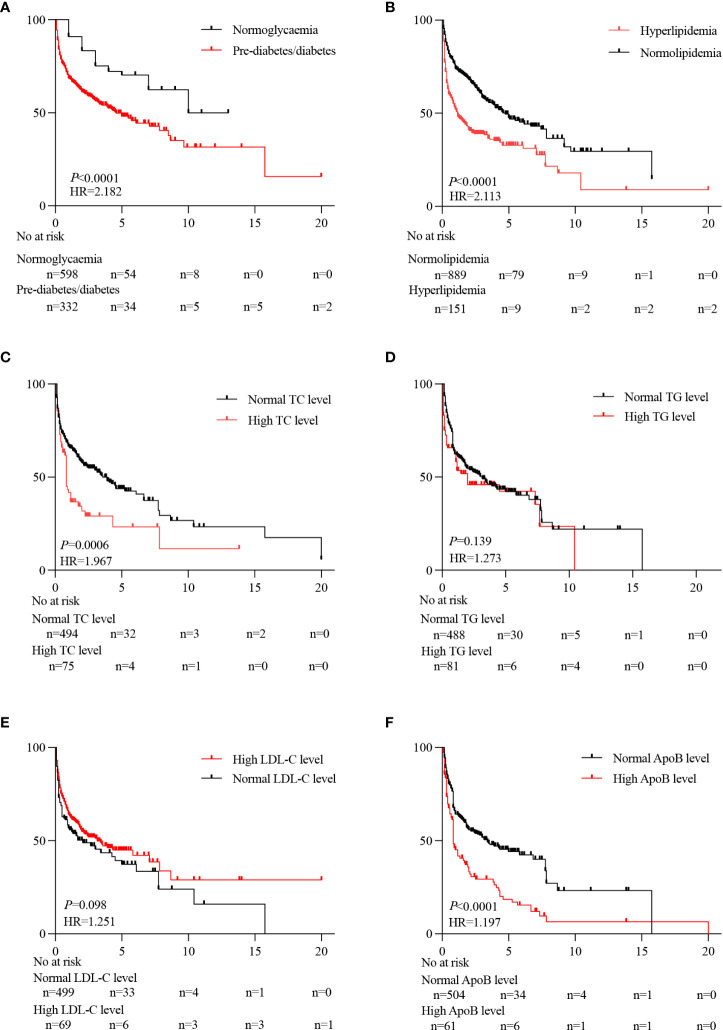
Effect of metabolic risk factors on the prognosis of PLC patients. Prognostic differences between pre-diabetes/diabetes and normoglycemia **(A)**. Prognostic differences between hyperlipidemia and normolipidemia **(B)**. Prognostic differences between normal TC level and high TC level **(C)**. Prognostic differences between normal TG level and high TG level **(D)**. Prognostic differences between normal LDL-C levels and high LDL-C levels **(E)**. Prognostic differences between normal ApoB level and high ApoB level **(F)**.

The average survival period of the PLC patients with normoglycaemia or pre-diabetes/diabetes was 10 or 4.58 years with a significant difference (*P* < 0.05) (**Figure 4A**). The average survival period of the PLC patients with normolipidemic or hyperlipidemia was 5.50 or 1.75 years with a significant difference (*P* < 0.05) (**Figure 4B**). The survival periods for the PLC patients with high TC or apolipoprotein B (ApoB) was 0.84 or 0.83 years, compared to the PLC patients with normal TC or ApoB which was 3.58 or 3.42 years (*P* < 0.05) (**Figures 4C–F**).

### Predictors for poor overall survival of PLC patients

Using univariate analysis, predictors for poor overall survival of PLC patients included: age (hazard ratio, 0.984 [95% confidence interval, 0.974–0.995], *P* < 0.05), gender ([1.574, 1.421–1.782], *P* < 0.05), MELD score ([1.051, 1.04–1.062], *P* < 0.05), ALBI score ([1.952, 1.778–2.143], *P* < 0.05), pALBI score ([2.843, 2.495–3.239], *P* < 0.05), ALT ([1.015, 1.008–1.024], *P* < 0.05), AST ([1.002, 1.001–1.002], *P* < 0.05), Tbil ([1.004, 1.003–1.004], *P* < 0.05), Alb ([0.939, 0.93–0.948], *P* < 0.05), platelet count ([1.002, 1.001-1.003], *P* < 0.05), neutrophil count ([1.13, 1.107–1.153], *P* < 0.05), monocyte count ([1.087, 1.008–1.172], *P* < 0.05), blood glucose ([1.952, 1.895–2.012], *P* < 0.05), LDL-C ([1.847, 1.46–2.278], *P* < 0.05), ApoB ([1.414, 1.121–1.783], *P* < 0.05), AFP ([1.931, 1.443–2.483], *P* < 0.05) (**Table 2**).

**Table 2:** Univariate and multivariate analysis of risk factors associated prognosis with PLC patients.

Parameter	Univariate analysis HR (95%CI)	*P* value	Multivariate analysis HR (95%CI)	*P* value
Age	0.984 (0.974-0.995)	<0.05		0.8
Gender: male/female	1.574 (1.421-1.782)	<0.05	2.094 (1.036-4.234)	<0.05
Cirrhosis	1.574 (1.014-1.973)	0.07		
PLC family history	3.465 (2.132-5.631)	0.1		
Smoking	0.99 (0.812-1.206)	0.05		
Alcohol	1.005 (0.807-1.251)	0.52		
Hypertension	1.064 (0.831-1.361)	0.07		
MELD score	1.051 (1.04-1.062)	<0.05		
ALBI score	1.952 (1.778-2.143)	<0.05		
pALBI score	2.843 (2.495-3.239)	<0.05		
ALT (IU/L)	1.015 (1.008-1.024)	<0.05	1.753 (1.086-2.832)	<0.05
AST (IU/L)	1.002 (1.001-1.002)	<0.05	1.066 (1.03-1.104)	<0.05
Tbil (μmol/L)	1.004 (1.003-1.004)	<0.05	1.005 (1.001-1.012)	<0.05
Alb (g/L)	0.939 (0.930-0.948)	<0.05	0.752 (0.57-0.993)	<0.05
Platelet count (× 10^9^/L)	1.002 (1.001-1.003)	<0.05		0.58
Neutrophil count (× 10^9^/L)	1.13 (1.107-1.153)	<0.05		0.06
Monocyte count (× 10^9^/L)	1.087 (1.008-1.172)	<0.05		0.39
Lymphocyte count (× 10^9^/L)	1.017 (0.96-1.032)	0.81		
Blood glucose (mmol/L)	1.952 (1.895-2.012)	<0.05	1.72 (1.064-2.265)	<0.05
TG (mmol/L)	0.983 (0.845-1.146)	0.08		
TC (mmol/L)	1.073 (0.988-1.167)	0.33	1.059 (1.017-1.102)	<0.05
LDL-C (mmol/L)	1.847 (1.46-2.278)	<0.05		0.09
ApoB (g/L)	1.414 (1.121-1.783)	<0.05	1.992 (0.366-5.826)	<0.05
AFP (ng/mL)	1.931 (1.443-2.483)	<0.05	2.213 (1.589-3.081)	<0.05

MELD, end-stage liver disease; ALBI, albumin-bilirubin; pALBI, platelet-albumin-bilirubin; ALT, alanine aminotransferase; AST, aspartate aminotransferase; Tbil, total bilirubin; Alb, albumin; TG, triglycerides; TC, total cholesterol; LDL-C, low-density lipoprotein cholesterol; ApoB, apolipoprotein B; AFP, alpha-fetoprotein.

To verify if these factors were significant in prediction for overall survival in PLC patients, multivariate analysis was applied. It was observed that the independent predictors included: gender (2.094 [1.036–4.234], *P* < 0.05), ALT ([1.753, 1.086–2.832], *P* < 0.05), AST ([1.066, 1.03–1.104], *P* < 0.05), Tbil ([1.005, 1.001–1.012], *P* < 0.05), Alb ([0.752, 0.57–0.993], *P* < 0.05), blood glucose ([1.72, 1.064–2.265], *P* < 0.05), TC ([1.059, 1.017–1.102], *P* < 0.05), ApoB ([1.992, 0.366–5.826], *P* < 0.05), AFP ([2.213, 1.589–3.081], *P* < 0.05) (**Table 2**).

## Discussion

The clinical importance of PLC, particularly in China, is due to the ~ 2.54-fold higher incidence than the world average (6). Although most Chinese PLC patients had HBV infection *via* the mother-fetal route, the incidence of PLC in adolescents is still rare (6, 22). It is reported that (23) incidence of HCC and age are directly correlated in most populations. In the current study, middle and older aged men between the ages of 50 ~ 70 years accounted for more than 80% of PLC patients, with the largest proportion of the PLC patients aged 50 ~ 60 years (28.86%). Therefore, it is necessary to monitor the onset of PLC within this age group carefully. Following determining the incidence of PLC patients, we found that the mean age of PLC patients increased from 52.74 ± 0.5 years in 2000 ~ 2004 to 58.63 ± 0.44 years in 2017 ~ 2020. Interestingly, there was the proportion of patients with BCLC grade A, Child-Turcotte-Pugh A class, and low MELD, ALBI, pALBI scores trended higher in this study, particularly the proportion of patients who underwent surgery or interventional treatment was significantly higher. In addition, there is an increasing trend of CHB-related HCC patients receiving antiviral therapy. These data suggest that such change may be due to more PLC patients having received advanced life-saving interventions over the past 20 years. These new conditions among PLC patients may be partially due to: The government organized vaccination against HBV program is being a common practice in Chinese communities over the last two decades, as well as the gradual availability of anti-viral medicines, which substantially reduce the disease progression towards cirrhosis and/or PLC. This is supported by our finding that there were 83.32% of PLC patients had HBV infection in the current study. Such observation suggests the good value of early intervention to reduce/prevent PLC in recent years. Therefore, we should strengthen the coverage of treatment for viral hepatitis patients to reduce the onset of PLC in the future.

There are significant sex differences in the incidence of PLC globally, showing that men are 2 ~ 4 times higher than women (24). Consistent with other reports from China and East Asia (23, 25, 26), we found that men accounted for most PLC patients in the current study (80.43%). Significant sex differences in the prognosis of HCC patients have been reported that females aged < 65 years had a better prognosis than males (25), supporting our current finding that the survival period of females was significantly longer than that of males, but the proportion of males was decreased by >10%. In our study, the percentage of female patients with elevated blood glucose or hyperlipidaemia increased from 12.5% to 21.24% or 14.29% to 23.9% from the period of 2005 ~ 2008 to the period of 2017 ~ 2020. The increased blood glucose and lipidemia contribute to increased incidence of NAFLD in female, and subsequent the prevalence of primary liver cancer. There was no linkage with age, may be due to relatively smaller in sample size. Classical risk factors for PLC, e.g., viral hepatitis, alcohol consumption, and smoking, are more common in males, but the risk factors of developing PLC in females are shifting from viral hepatitis to NAFLD at the same period (27). Such finding suggests that women with NAFLD, especially those over the age of 50, are more likely to develop advanced fibrosis than men (28). Although CHB was still the predominant cause of PLC (83.32%) in the current study, the proportion of non-hepatitis virus-related PLC patients was also increased ~ 10 fold over the last 20 years, suggesting the critical potential influence of metabolism-related liver disease for the PLC development. Thus, we should enhance screening or/and early intervention for liver cancer progress in female patients with non-viral chronic hepatic diseases.

AFP remains an important biomarker for the diagnosis of PLC in East Asia. Serum AFP expression levels are elevated in many PLC patients, and persistently elevated AFP levels are often associated with PLC progression and relapse (29). However, AFP < 20ng/mL (AFP-negative) has been found in the current study and many PLC clinical research (29, 30), which implies that AFP can’t be used to screen PLC in these cases. Future studies should be carried out to find reliable biomarkers for diagnosing AFP-negative PLC. Thus, ensuring the timely initiation of treatment.

Long-term ALT abnormalities are strongly associated with the risk of developing HCC (31, 32). This is in line with our finding that the mortality was 16.85% or 31.46% of PLC patients with ALT between 40 ~ 60 IU/L or ALT ≥ 60 IU/L. The PLC patients with ALT ≥ 40 IU/L had a higher risk of HCC progression than those PLC patients with ALT 40 ~ 60 IU/L. This is further supported by a South Korean team (33) showing that the risk of liver-related mortality is increased even in patients with high normal ALT levels (ALT 35 ~ 45 IU/L). However, the definition of ULN of ALT is not consistent in the different regions, the ULN of ALT used in our institute is 64 IU/L, which is higher than the ULN in the study of the East Asian populations mentioned above (33, 34), and the prognosis of PLC patients with ALT < 40 IU/L was better than that of the patients with ALT ≥ 40 IU/L, showing that ALT is inversely correlated with survival time in PLC patients. Our data is similar to other reports (35), suggesting that the current standards of China may make it difficult to accurately assess the monitoring of PLC risk. Thus, these data suggest that lowering ALT properly improves the prognosis of PLC patients.

Because the liver is the main organ in regulating sugar and lipid metabolism, compromised liver function from PLC patients may contribute to the imbalance of glucose and lipid composition, and ultimately promotes PLC progression due to the development of prediabetes/diabetes and hyperlipidaemia (36–38). This is supported by the report (39), showing that there is ~ 100-fold increased risk of developing PLC in patients with Diabetes Mellitus compared to those without. In the current study, the percentage of PLC patients with comorbid glucose abnormalities was increased ~ 5-fold over the last two decades, accompanied by significantly reduced survival time, compared to that of normoglycemic PLC patients. We also found that blood glucose ≥ 5.5 mmol/L was a significant risk factor affecting the prognosis of PLC patients. Thus, such information highlights the seriousness of blood glucose levels involved in the risk of PLC progression, and it is fundamentally important to control blood glucose among these PLC patients for the best of their prognosis and outcomes.

Moreover, we found that there was an inverse correlation between the proportion of PLC patients with hyperlipidaemia and the survival period. Our data is in line with a Japanese team (40), showing that increased incidence of non-hepatitis virus-related HCC patients with diabetes mellitus and hyperlipidaemia over the last two decades, as well as, metabolic factors are non-negligible risk factors for HCC. Furthermore, an inverse correlation between the survival rate of hepatitis virus-related PLC patients and hyperlipidaemia (41). Therefore, it is absolutely necessary to strictly manage lipid levels in PLC patients to minimize the risk for further PLC progression.

Furthermore, in the current study, the prognosis of PLC patients with hyperlipidaemia was poorer than that of PLC patients with normal lipids. This is consistent with Hwang et al. (42) showing that TC could be used as a marker to predict PLC recurrence because TC is correlated well with serum AFP. Moreover, AFP levels are positively correlated with poor prognosis of patients with PLC (43). Thus, in addition to AFP, TC may be a potential adjunctive in assessing the prognosis of PLC patients. Interestingly lipoproteins promote the proliferation and invasion of cancer cells, but also enhance anti-tumor immunity (44). ApoB is associated with tumour size and poor patient prognosis (45). ApoB/ApoA-I rates in HCC patients are correlated with AFP, distant metastases, and TNM stage, particularly in the patients with high ApoB/ApoA-I rates (46). Thus, managing blood lipids is equally as important as managing TC and ApoB to facilitate more accurate prognostic assessment in PLC patients.

Although we described the real-life clinical experience of a busy general hospital, the results reliably reflect the clinical features and treatment approaches to PLC in China over the past 20 years. There are some limitations in the current study. Most PLC patients were CHB-related HCC in the current study, other causes will also be included, such as diabetes mellitus, and other metabolic syndrome-related biomarkers, such as body mass index (BMI), should be explored in the future. The epidemiological characteristics of HCC vary considerably across geographic regions and ethnic groups. The epidemiological characteristics of HCC vary considerably across geographic regions and ethnic groups. However, our current study was a single-center focused investigation, which will be extended to multicenter with different regions/ethnic backgrounds in the future.

In conclusion, we found that the age of the PLCs, female patients, and non-viral hepatitis-related PLC incidence gradually increased. Therefore, we should monitor PLC patients with AFP-negative regularly and discover novel biological markers to assess PLC progression. Proper control of glucose/lipids or ALT is beneficial for PLC patients to improve their prognosis. Such information offers some evidence for the management and prediction of PLC for clinicians and public health sections.

## Data availability statement

The raw data supporting the conclusions of this article will be made available by the authors, without undue reservation.

## Ethics statement

The studies involving human participants were reviewed and approved by the Human Ethics Committee, Ruijin Hospital, Shanghai Jiao Tong University School of Medicine. Written informed consent for participation was not required for this study in accordance with the national legislation and the institutional requirements.

## Author contributions

HW was fully responsible for the conduct of this study. HW and YD designed the experiment. YD, MF, DM, and GZ coordinated the study. YD performed the majority of the experiment and drafted the manuscript. SB & HW & KL, interpreted data and revised the manuscript. Clinical data collection was completed by MF, DM, GZ, XW, BA, YX, SL, LL and QX. All authors contributed to the article and approved the submitted version.

## Glossary

**Table:**

PLC	primary liver cancer
HCC	hepatocellular carcinoma
HBV	hepatitis B virus
HCV	hepatitis C virus
NAFLD	non-alcoholic fatty liver disease
NAs	nucleos(t)ide analogs
DAAs	direct-acting antivirals
AIH	autoimmune hepatitis
PBC	primary biliary cholangitis
DILI	drug induced liver injury
ALD	alcoholic liver disease
PSC	primary sclerosing cholangitis
PLD	parasitic liver disease
AASLD	the American Association for the Study of Liver Diseases
TC	total cholesterol
LDL-C	low-density lipoprotein cholesterol
TG	triglycerides
ApoB	apolipoprotein B
BCLC stage	the Barcelona Clinic Liver Cancer stage
MELD	end-stage liver disease
ALBI	albumin-bilirubin
pALBI	platelet-albumin-bilirubin
ICC	intrahepatic cholangiocarcinoma
AFP	alpha-fetoprotein
AST	aspartate aminotransferase
Tbil	total bilirubin
Alb	albumin
ALT	alanine aminotransferase.
