# Supplementary material for: The 20 years transition of clinical characteristics and metabolic risk factors in primary liver cancer patients from China
Source: Front Oncol. 2023 Mar 14;13:1109980. doi: 10.3389/fonc.2023.1109980 (PMC10043326; doi:10.3389/fonc.2023.1109980)
Supplement: Supplementary file 1 [file DataSheet_1.docx]

**The 20 years transition of clinical characteristics and metabolic risk factors in primary liver cancer patients from China**


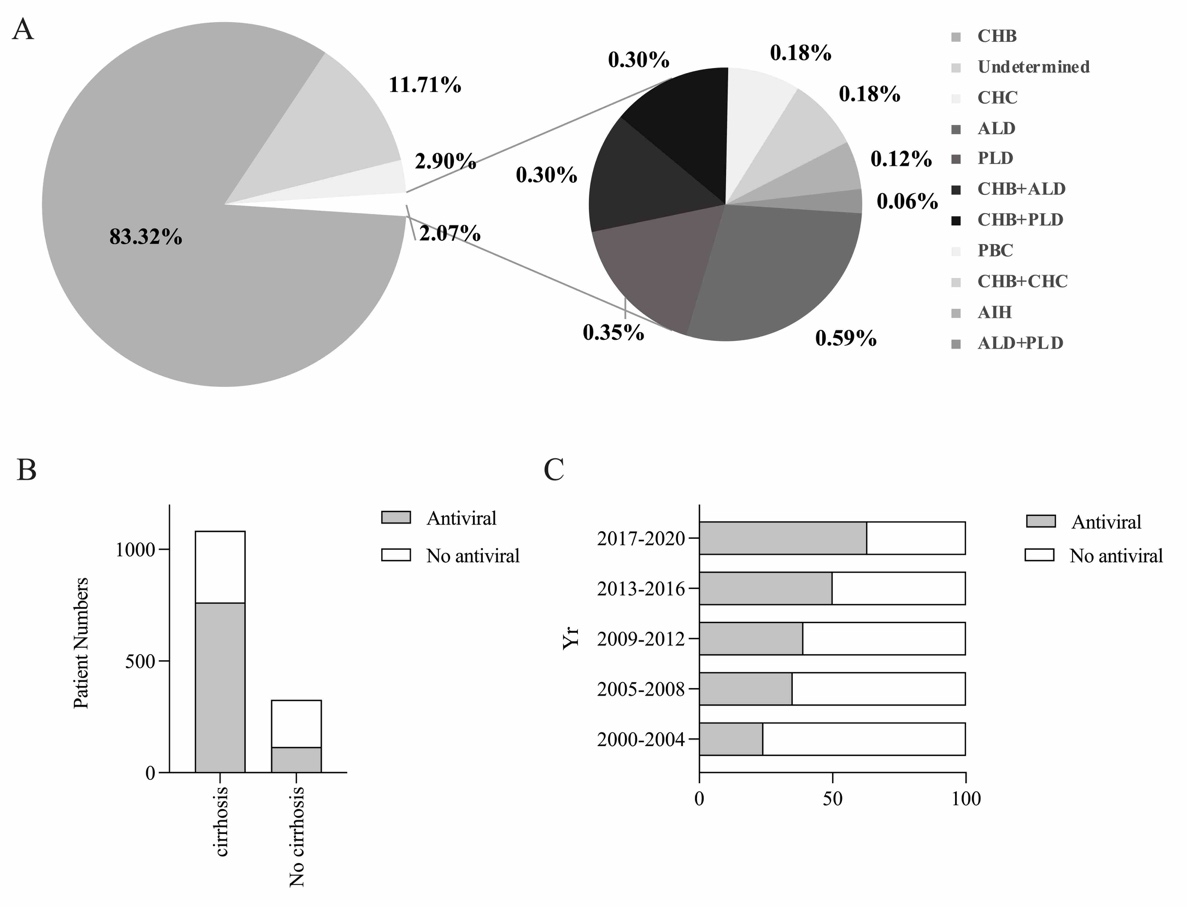


Fig. S1. Underlying diseases of PLC patients and changes in the proportion of antiviral therapy in CHB-related PLC patients. Underlying diseases of PLC patients (A). Percentage of cirrhotic *vs* non-cirrhotic CHB-related PLC patients receiving antiviral therapy (B). Change in the proportion of CHB-related PLC patients receiving antiviral therapy (C).


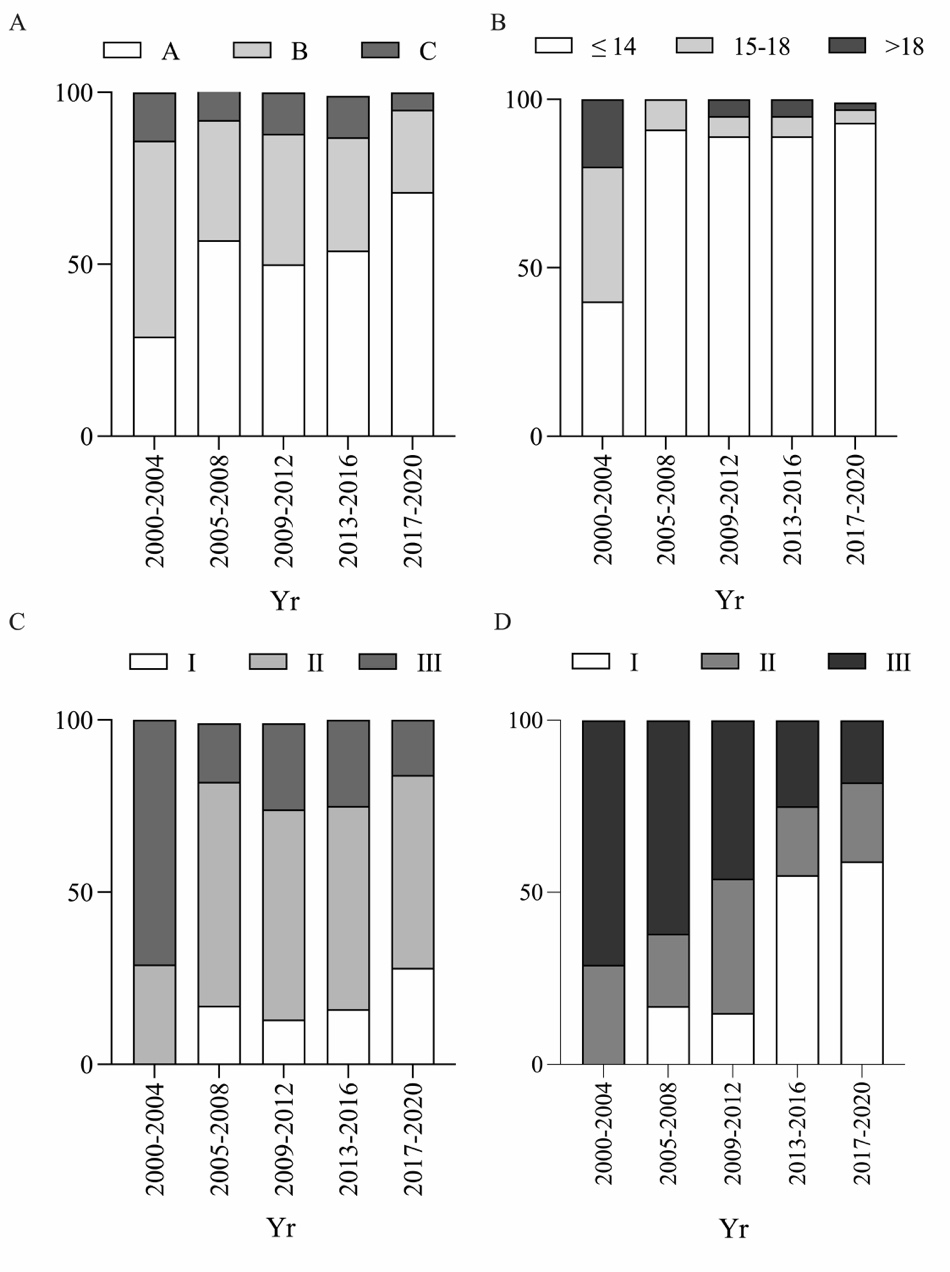


Fig. S2. Changes in the percentage of different subgroups among PLC patients. Change in proportion of different Child-Pugh classifications (A). Change in proportion of different MELD scores (B). Change in proportion of different ALBI grades (C). Change in proportion of different pALBI grades (D).

**ALT stratification among PLC patients and related prognosis**

Following stratification of the percentage of these patients based on the alanine transaminase (ALT), there were 267 (15.79%), 607 (35.9%), 285 (16.85%) or 532 (31.46%) PLC patients in ALT < 20 IU/L, 20 ~ 40 IU/L, 40 ~ 60 IU/L or > 60 IU/L, respectively (Fig. S3). Among 1183 PLC patients with cirrhosis, 184 (15.55%), 468 (39.56%), 218 (18.43%) or 313 (26.46%) patients had ALT < 20 IU/L, 20 ~ 40 IU/L, 40 ~ 60 IU/L, or > 60 IU/L respectively.


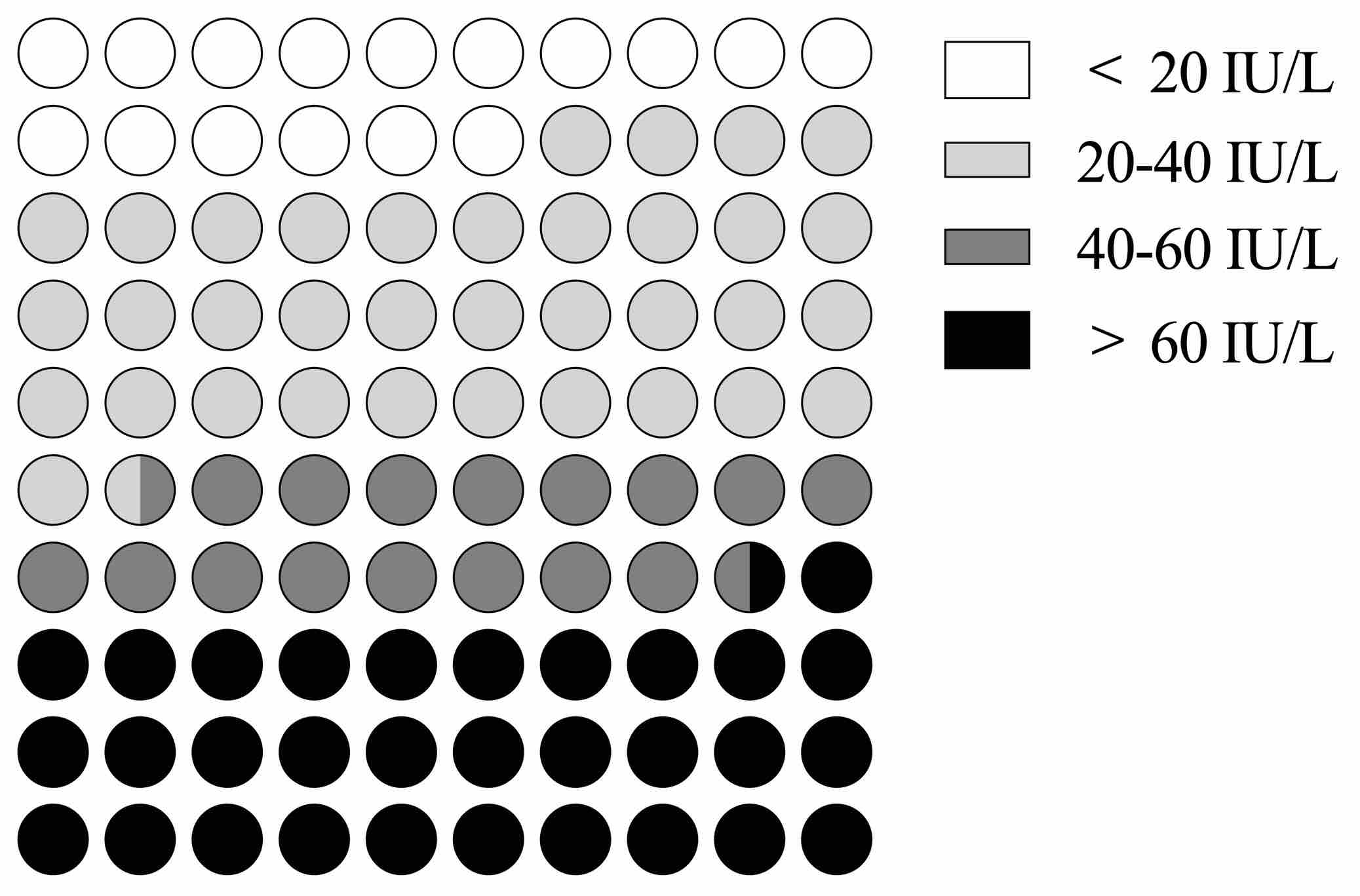


Fig. S3. ALT stratification among PLC patients

The survival period of patients with ALT ≥ 40 IU/L was significantly shorter than those of patients with ALT < 40 IU/L among PLC patients with/without cirrhosis (*P* < 0.0001) (Fig. S4A-B).

The average survival periods of the male or female PLC patients were 3.33 or 4.92 years (*P* < 0.05) (Fig. S4C). The average survival period of the PLC patients with or without diabetes mellitus was 3.92 or 4 years with no significant difference (*P* > 0.05) (Fig. S4D).


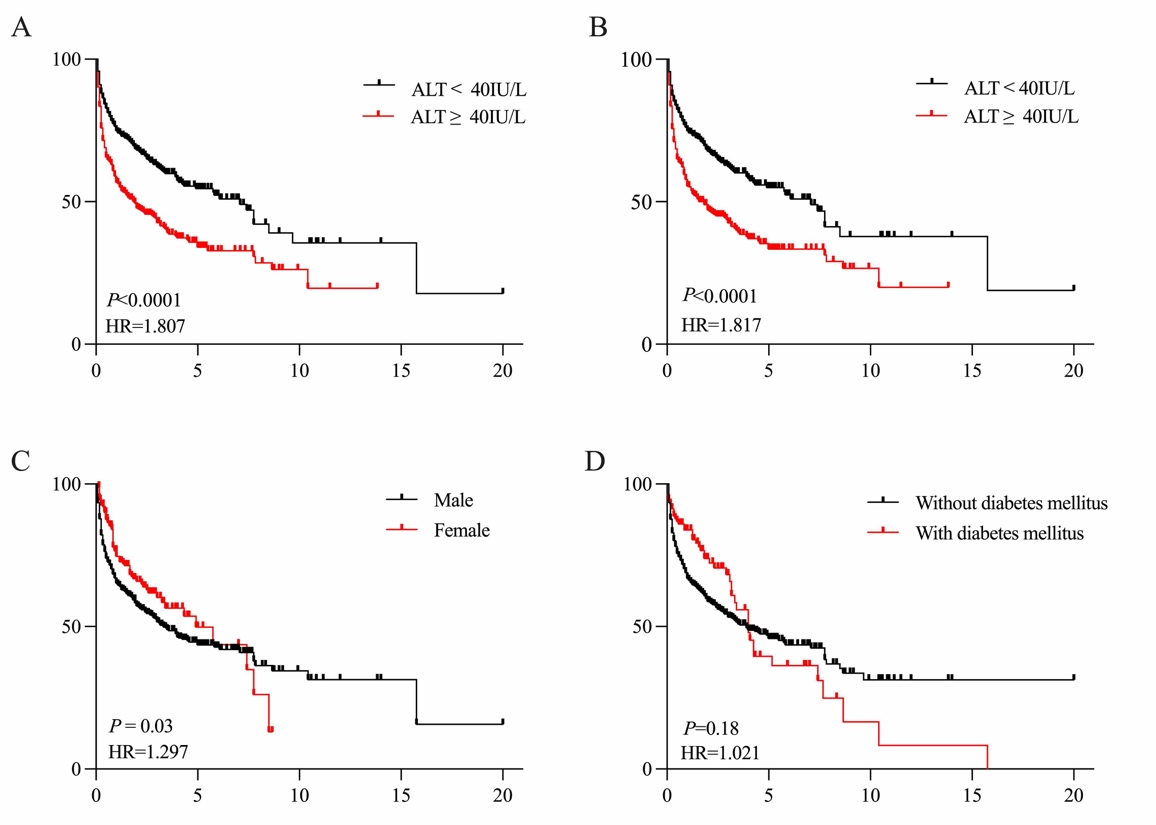


Fig. S4. Prognosis of PLC patients under different subgroups. Prognosis of ALT ≥ 40 IU/L *vs* ALT < 40 IU/L among the cirrhotic group (A). Prognosis of ALT ≥ 40 IU/L *vs* ALT < 40 IU/L among the non-cirrhotic group (B). Prognosis of male vs female patients (C). Prognosis of patients with/without a history of diabetes mellitus(D).
